# Effect of Ionic Radius in Metal Nitrate on Pore Generation of Cellulose Acetate in Polymer Nanocomposite

**DOI:** 10.3390/polym12040981

**Published:** 2020-04-23

**Authors:** Woong Gi Lee, Younghyun Cho, Sang Wook Kang

**Affiliations:** 1 Department of Chemistry, Sangmyung University, Seoul 03016, Korea; even004@naver.com; 2 Department of Energy Systems Engineering, Soonchunhyang University, Asan 31538, Korea; 3 Department of Chemistry and Energy Engineering, Sangmyung University, Seoul 03016, Korea

**Keywords:** cellulose acetate, porosity, ionic radius, water-pressure

## Abstract

To prepare a porous cellulose acetate (CA) for application as a battery separator, Cd(NO_3_)_2_·4H_2_O was utilized with water-pressure as an external physical force. When the CA was complexed with Cd(NO_3_)_2_·4H_2_O and exposed to external water-pressure, the water-flux through the CA was observed, indicating the generation of pores in the polymer. Furthermore, as the hydraulic pressure increased, the water-flux increased proportionally, indicating the possibility of control for the porosity and pore size. Surprisingly, the value above 250 LMH (L/m^2^h) observed at the ratio of 1:0.35 (mole ratio of CA: Cd(NO_3_)_2_·4H_2_O) was of higher flux than those of CA/other metal nitrate salts (Ni(NO_3_)_2_ and Mg(NO_3_)_2_) complexes. The higher value indicated that the larger and abundant pores were generated in the cellulose acetate at the same water-pressure. Thus, it could be thought that the Cd(NO_3_)_2_·4H_2_O salt played a role as a stronger plasticizer than the other metal nitrate salts such as Ni(NO_3_)_2_ and Mg(NO_3_)_2_. These results were attributable to the fact that the atomic radius and ionic radius of the Cd were largest among the three elements, resulting in the relatively larger Cd of the Cd(NO_3_)_2_ that could easily be dissociated into cations and NO_3_^−^ ions. As a result, the free NO_3_^−^ ions could be readily hydrated with water molecules, causing the plasticization effect on the chains of cellulose acetate. The coordinative interactions between the CA and Cd(NO_3_)_2_·4H_2_O were investigated by IR spectroscopy. The change of ionic species in Cd(NO_3_)_2_·4H_2_O was analyzed by Raman spectroscopy.

## 1. Introduction

Nanoporous materials have received much attention for their possibilities of being utilized for gas separation, catalysis, battery separators, medicine, and water treatment [1,2]. Currently, the preparation or synthesis of nanoporous materials has been explosively explored due to showing various and unique characteristics. For instance, the Lei Qian group suggested a special method to generate nanoparticles into porous materials with the evaporation phenomena of solvents [3]. On the other hand, the Dubinsky group showed that polymerization-induced phase separation could generate the hybrid porous material [4]. Recently, unique structures such as porous polymer networks (PPNs), porous organic polymers (POPs), metal-organic frameworks (MOFs) and porous aromatic frameworks (PAFs) have been investigated due to their gas storage characteristics [5,6,7,8]. On the other hand, Yiming et al. reported enhanced CO_2_ uptake through microporous carbon material [9]. Additionally, Atsushi Takase et al. evaluated the SF_6_ and N_2_ separation ability of nanoporous carbons using an adsorption mechanism [10]. Moreover, porous materials as separators to improve the efficiency of batteries have been receiving attention. Specifically, porous organic materials have made promising candidates for highly efficient battery separators due to their easily tunable morphology by using smart functional materials and chemistry [11,12,13,14]. In detail, the lithium ion battery separator was prepared generally with polymers such as polyethylene (PE) or polypropylene (PP) because of their attractive physical and electrochemical stability. However, PP and PE separators have some drawbacks, which would show low wettability, resulting in the low ionic conductivity. Furthermore, low thermal stability played a role as an obstacle for practical applications. To overcome these drawbacks, porous inorganic material, nanoparticle, high thermal-resistance polymer and separator coated thermal material have been used as separator [15,16,17]. Especially, Meinan et al. developed the thermally stable separator with high performance by using the pure inorganic material [18]. Furthermore, the Lee group reported the thermally stable polyimide nanofiber with Al oxide particles as separator [19].

To date, porous materials have been formed through a variety of methods such as thermally induced phase separation, evaporation induced phase separation, phase inversion and track-etching [20,21,22]. For example, Jian Zhao et al. proposed a method to fabricate microporous material using the solvent evaporation-including separation. This method used a ternary solution, such as a liquid silicone rubber precursor of liquid paraffin and hexane, and this solution was cast to form a film. When the hexane and liquid paraffin was removed, micropores from less than 300 to over 1200 µm were generated in the silicone rubber film [23]. Hermsdorf et al. suggested the fabrication of porous polyimide film onto a silicon support coated with an oxide layer. Polyimide membranes are used as a starting material for a sample preparation based on the high energy of ion irradiation. As a result, the pore size in the membrane was an average of 73 nm and the pore shape was conical [24]. However, some problems of these methods are the complications and the high-cost of the process. To solve these drawbacks, our group reported the method to fabricate pores using the ionic liquid and solvated inorganic complex [25,26]. The nickel nitrate (Ni(NO_3_)_2_·6H_2_O) as inorganic complex was well dissolved in water or acetone and it can be applied to cellulose acetate solution dissolved acetone/water (w/w 8/2). As a result, the pore size and volume of the polymer matrix increased by water pressure and the pores were uniformly distributed [26]. In this study, we suggested the environment-friendly and energy-efficient method to synthesize the pores, which is close to the straight type in a cellulose acetate (CA) polymer matrix [27], using a combination of inorganic complex (Cd(NO_3_)_2_·4H_2_O) and isostatic water pressure. Especially, we investigated the effect of the ionic radius in metal nitrate on both the pore generation and pore control of cellulose acetate.

## 2. Experimental

Separator fabrication: polymer (cellulose acetate, Sigma-Aldrich Co. Milwaukee, WI, USA) was dissolved into acetone/water co-solvents. Then, cadmium (II) nitrate hexahydrate (Cd(NO_3_)_2_·4H_2_O, Sigma-Aldrich Co. Milwaukee, WI, USA) was incorporated into polymer solution by the mole ratio of the Cd salts to the monomeric unit of polymer. The prepared mixture was stirred for one hour at room temperature, and then casted for the free-standing film on the plate. Finally, the free-standing films were dried at atmosphere pressure for 30 min. The dried polymer films with Cd(NO_3_)_2_·4H_2_O were placed into cells such that water molecules could be passed through the composite films. Then, the various water-pressures ranging from 2 to 8 bar were applied to the cells equipped with the polymer composites containing Cd(NO_3_)_2_·4H_2_O. The water flux of the films for varying porosity was measured and expressed as L/m^2^h.

## 3. Results and Discussion

**Figure 1:**
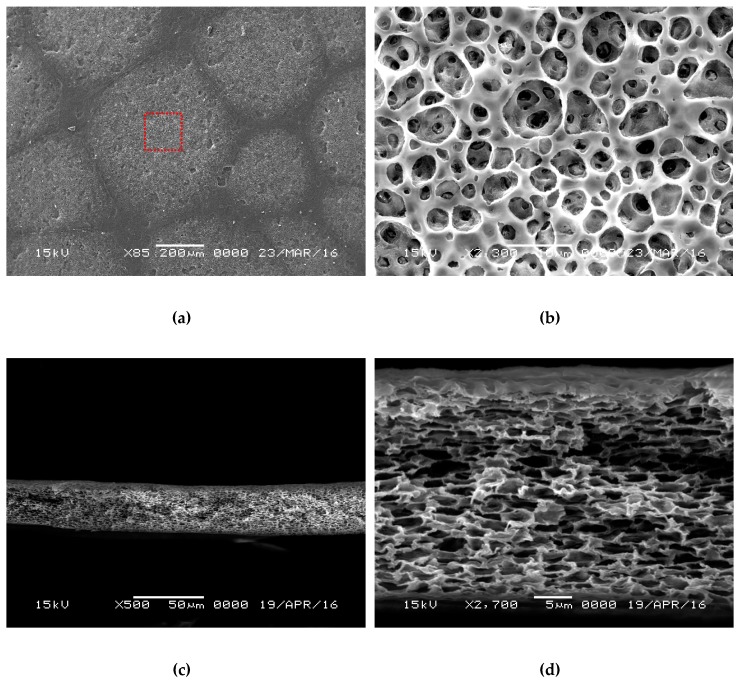
SEM images: (**a**) plane view of porous CA polymer exposed to external water-pressures (scale bar = 200 μm), (**b**) the enlarged red-dot square in plane view (scale bar = 10 μm), (**c**) cross-section view (scale bar = 50 μm) and (**d**) enlarged view of cross-section (scale bar = 5 μm).

Scanning electron microscopy (SEM JSM-5600LV, JEOL, Tokyo, Japan) was utilized to observe the pore formation on and in polymers with the Cd(NO_3_)_2_·4H_2_O additive after being exposed to external water-pressures. Figure 1a,b represents the surface morphology of the CA matrix which was dissolved in acetone/water co-solvents with the Cd(NO_3_)_2_·4H_2_O additive. The SEM image clearly exhibited that there were abundant pores on the surface when the CA polymer with Cd(NO_3_)_2_·4H_2_O was exposed to water-molecules, while the pores were not observed in the neat CA polymer even when the water-pressure was applied as in a previous study [28]. Furthermore, the cross-section images of the CA matrix containing the Cd(NO_3_)_2_·4H_2_O additive shown in Figure 1c,d indicate that that pores were well fabricated in the polymers. In contrast, when the neat CA was dissolved in pure acetone, there were no pores on the surface of the polymer. In order to precisely control the pore size and porosity, we introduced the new method to utilize the Cd(NO_3_)_2_·4H_2_O in acetone/water co-solvent. The resulting SEM image clearly implies that the pore size and porosity of polymer matrix could be controllable by inorganic salts and water-molecules. It was noted that there were two major plausible explanations for these phenomena: (1) the Cd(NO_3_)_2_·4H_2_O aggregates solvated in the polymer matrix during the solidification while the volatile acetone rapidly evaporated, resulting in the remaining Cd(NO_3_)_2_·4H_2_O aggregates forming well-defined pores in the CA polymer matrix; or (2) that there were ionic associations between the ions generated from the Cd(NO_3_)_2_ and the water molecules. These strong interactions delayed the evaporation of the water molecules in the CA polymer matrix, resulting in the formation of pores on the surface.

**Figure 2:**
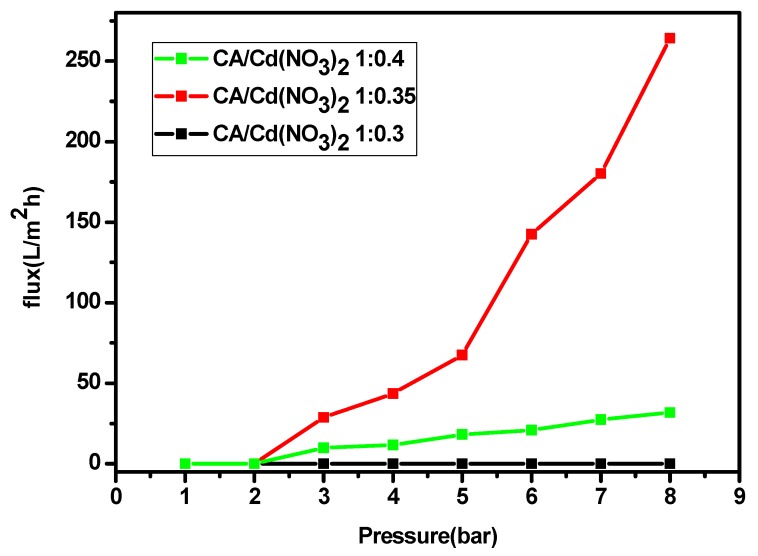
Flux measured through neat CA and CA with Cd(NO_3_)_2_·4H_2_O at various water pressures.

The water flux through the porous CA polymer containing different ratios of Cd(NO_3_)_2_·4H_2_O is shown in Figure 2. The water flux was not observed up to 2 bar, indicating that pores in CA polymer were not formed. However, the water flux gradually increased for 1:0.35 and 1:0.40 mol ratios CA/Cd(NO_3_)_2_·4H_2_O polymer matrix at 3 bar. An explanation for these phenomena is that the water-channel was generated through weakened regions in the CA polymer, resulting in the observation of water-flux. However, the flux of neat CA polymer was not observed up to 7-bar water pressure and the low flux of 2.55 L/m^2^h was observed only in the 8-bar water pressure [26]. From these results, it was thought that the CA polymer without Cd(NO_3_)_2_·4H_2_O was confirmed not to have pores capable of being penetrated for water molecules through the polymer chains. Even though the amounts of Cd(NO_3_)_2_·4H_2_O beyond the aforesaid ratios could be added, the increase in flux was not observed since the interactions between polymer chains were strengthened by crosslinking phenomena in CA polymer chains. Therefore, the best performance capable of being easily controllable for pores was the ratio of 1:0.35 for CA/Cd salts. Especially, the value above 250 LMH (L/m^2^ h) observed at the ratio of 1:0.35 was higher flux than those of CA/other metal nitrate salts (Ni(NO_3_)_2_ and Mg(NO_3_)_2_) complexes. The higher value indicated that the larger and abundant pores were generated in the cellulose acetate with the same physical external forces. Thus, it could be thought that Cd(NO_3_)_2_·4H_2_O salt played a role as a stronger plasticizing agent than the other metal nitrate salts such as Ni(NO_3_)_2_ and Mg(NO_3_)_2_.

Table 1 showed the comparison of atomic radius and ionic radius of Cd, Ni and Mg utilized in previous with this study to use Cd salts. As shown in Table 1, the atomic radius and ionic radius of Cd was largest among the three elements. Thus, it was thought that the Cd(NO_3_)_2_ that had relatively larger Cd could easily be dissociated into Cd ions and NO_3_^−^ ions. As a result, the free NO_3_^−^ ions could be readily hydrated with water molecules, resulting in the plasticization effect on the chains of cellulose acetate. Therefore, when the cellulose acetate containing the Cd(NO_3_)_2_ salts were exposed to high-intensive water-pressure, the pores could be generated more easily than in the other complexes containing Ni or Mg salts.

**Figure 3:**
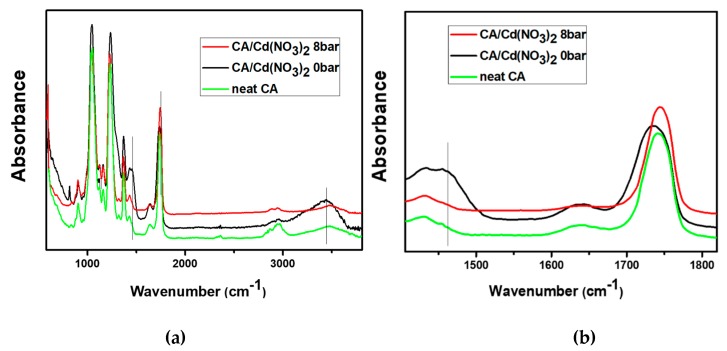
FT-IR spectra of neat CA and 1/0.35 CA/Cd(NO_3_)_2_·4H_2_O polymer matrix at 0 bar and 8 bar water pressures: (**a**) total range and (**b**) enlarged specific region.

To understand the role of Cd(NO_3_)_2_·4H_2_O solvates during the water pressure process, we performed Fourier transform infrared (FT-IR) spectra. Figure 3a shows the IR spectra of pristine polymer and 1/0.35 CA/Cd(NO_3_)_2_·4H_2_O from 0 bar and 8 bar water pressures, respectively. The pristine polymer in IR spectra was observed at 3500 cm^−1^, corresponding to the hydroxyl group of the CA polymer. In contrast, the CA polymer matrix with Cd(NO_3_)_2_·4H_2_O showed the absorption bands for CA/Cd(NO_3_)_2_·4H_2_O at 3400 cm^−1^, resulting as the abundance of water molecules for Cd(NO_3_)_2_·4H_2_O caused the intensity of the OH adsorption peak to be increased. The CA/Cd(NO_3_)_2_·4H_2_O composites exposed to 8 bar were observed at the 3400 cm^−1^, which decreased in intensity and shifted to 3500 cm^−1^, indicating that a considerable amount of Cd(NO_3_)_2_·4H_2_O was removed by high water-pressure process. The peak observed at the 1748 cm^−1^ as shown Figure 3b was shifted to 1731 cm^−1^ for polymer containing Cd(NO_3_)_2_·4H_2_O. This result indicated that the carbonyl group of polymers interacted with the Cd ion. When the polymer matrix containing CA/Cd(NO_3_)_2_·4H_2_O was exposed to external physical forces, most of the Cd(NO_3_)_2_·4H_2_O was removed from the polymer matrix and recovered to 1748 cm^−1^. Furthermore, the peak observed at the 1460 cm^−1^ as shown in Figure 3b disappeared when the polymer was exposed to external physical forces. It was also thought that the Cd(NO_3_)_2_·4H_2_O was removed by physical pressure and the peak of 1460 cm^−1^ disappeared.

**Figure 4:**
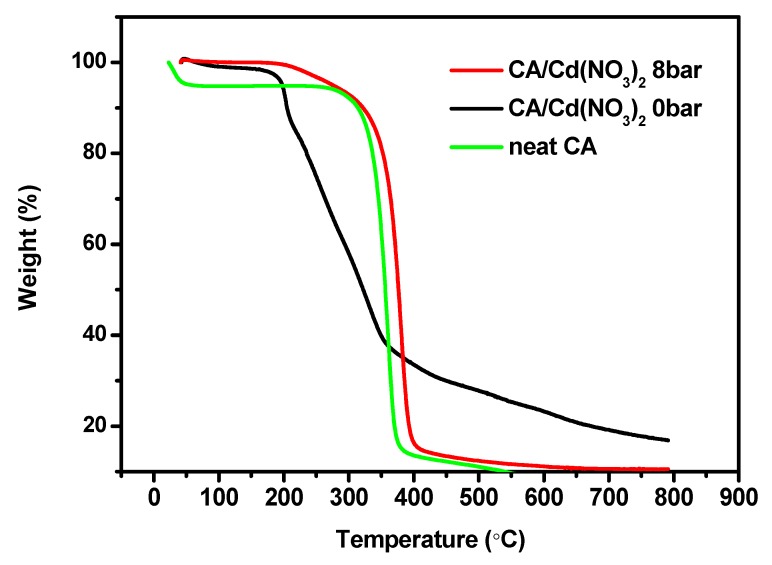
TGA analysis of neat CA and CA/Cd(NO_3_)_2_·4H_2_O polymer matrix at 0 bar and 8 bar.

The thermal degradation pattern for polymers was observed by thermogravimetric analysis (TGA). Figure 4 showed that most of the pristine polymer and CA with Cd(NO_3_)_2_·4H_2_O exposed at 8 bar were decomposed at around 300 °C. On the other hand, about 60 wt % of polymer with Cd(NO_3_)_2_·4H_2_O at 0 bar was degraded at between 200 to 350 °C, and 40 wt % was degraded at between 350 to 800 °C. Since the boiling point of Cd(NO_3_)_2_·4H_2_O was known to be 132 °C, it was thought that the chains in polymer were loosened by solvated cadmium nitrate. Thus, the loss of about 60 wt % CA with Cd(NO_3_)_2_·4H_2_O was attributable to the degradation of solvated Cd(NO_3_)_2_·4H_2_O and loosened polymer chain. However, the thermal stability of the polymer matrix increased after water-pressure treatment. The change in the thermal stability was generated as most of the Cd(NO_3_)_2_∙4H_2_O were removed in the polymer. As a result, the thermal stability of the polymer matrix (with Cd(NO_3_)_2_·4H_2_O removed) by water-pressure treatment was similar to the neat polymer matrix.

**Figure 5:**
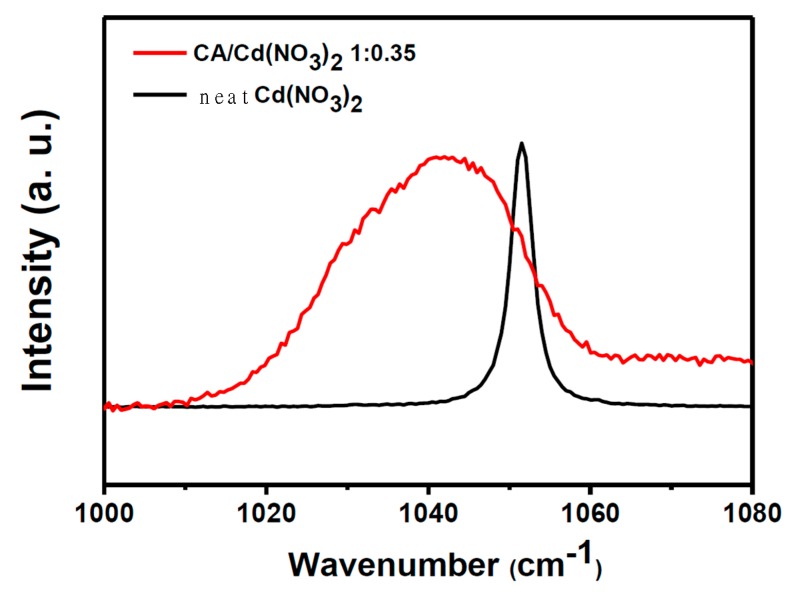
Raman spectra of neat Cd(NO_3_)_2_·4H_2_O and CA/Cd(NO_3_)_2_·4H_2_O polymer matrix.

To investigate the ionic species such as ion aggregates, ion pairs and free ions in NO_3_^−^ ions for pristine Cd(NO_3_)_2_ and Cd(NO_3_)_2_ in the polymer, Raman spectra were acquired as shown in Figure 5. Since the ionic species of NO_3_^−^ at 1034 (free ions), 1040 (ion pairs) and 1045 (ionic aggregates) cm^−1^ was observed, the spectra were focused in the range of 1000 to 1080 cm^−1^. As shown in Figure 5, the peak of pristine Cd(NO_3_)_2_ was observed at 1051.5 cm^−1^.

**Figure 6:**
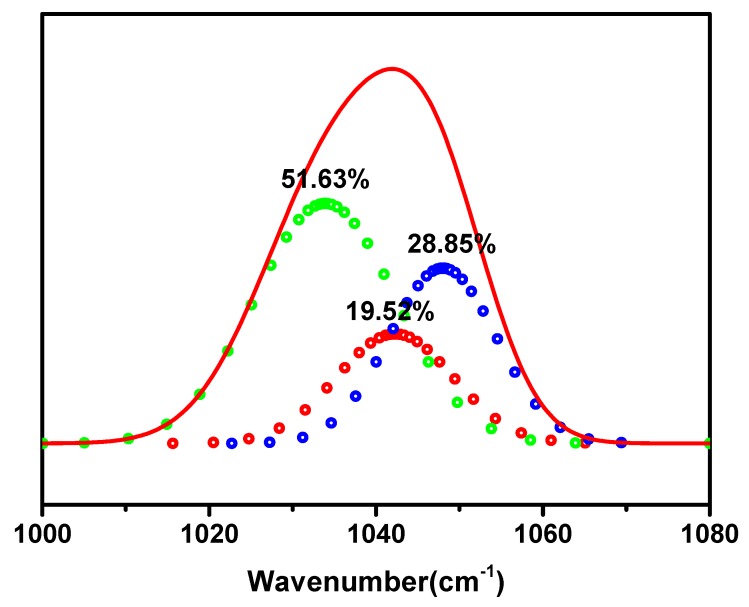
Deconvoluted Raman spectra for NO_3_^−^ in the CA/Cd(NO_3_)_2_·4H_2_O composite.

When Cd(NO_3_)_2_ was incorporated to the polymer, the peak was shifted from 1051.5 to 1040 cm^−1^ and was broadened as shown Figure 5, indicating that the Cd(NO_3_)_2_ incorporated into the CA polymer existed as free ions and ion pairs as well as aggregates. In the case of the Cd(NO_3_)_2_ incorporated into the CA, the relative percentages for various ionic species were shown in Figure 6. Surprisingly, new peaks were observed at 1034.2 and 1045.6 cm^−1^, indicating the free ions and ion aggregates. Deconvoluted results were shown for each of the regions in Figure 6.

Based on these results, the Cd(NO_3_)_2_ incorporated into the polymer existed as abundant free ions in the polymer chain as shown in Table 2.

## 4. Conclusions

**Scheme 1:**
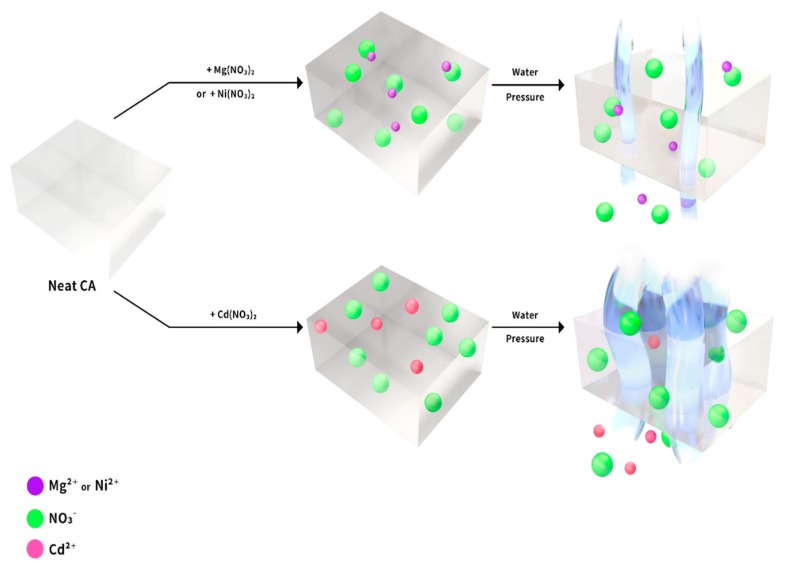
Comparison of pore generation by species of metal salts in cellulose acetate.

A porous polymer matrix was successfully fabricated by Cd(NO_3_)_2_·4H_2_O and external physical forces as shown in Scheme 1. When water pressure as physical force was applied to the polymer matrix, pore size gradually increased. Furthermore, it was observed the water flux increased as the water pressure increased. These results indicated that the water molecule that penetrated into the polymer caused the chains to be weakened by solvated Cd(NO_3_)_2_·4H_2_O. Surprisingly, most of Cd(NO_3_)_2_·4H_2_O in the polymer was extracted by the external forces. Furthermore, the relationship between radius characteristics in the metal salts and pore generation in the polymer was also investigated. It was found that since Cd(NO_3_)_2_ had relatively bigger Cd, it could easily be dissociated into Cd ions and NO_3_^−^ ions and the free NO_3_^−^ ions could be readily hydrated with water molecules, resulting in the plasticization effect on the chains of cellulose acetate. Therefore, when the cellulose acetate containing Cd(NO_3_)_2_ salts were exposed to high-intensive water-pressure, the pores could be more easily generated than other complexes containing Ni or Mg salts. Thus, these results were expected to be utilized for designing porous materials with specific pore-sizes.

## Figures and Tables

**Table 1:** Atomic radius and ionic radius of various elements utilized.

Elements	Atomic Radius (pm)	Ionic Radius (pm)
Cd	155	109
Ni	135	83
Mg	150	86
Zn	135	88

**Table 2:** Comparison of free ions, ion pair, and ionic aggregates % for NO_3_^−^ in Cd(NO_3_)_2_ in CA.

	Free ions %	Ion Pair %	Ionic Aggregates %
Cd(NO_3_)_2_ in CA	51.63%	19.52%	28.85%
